# Contextualised digital health communication infrastructure standards for resource-constrained settings: Perception of digital health stakeholders regarding suitability for Uganda’s health system

**DOI:** 10.1371/journal.pdig.0000603

**Published:** 2024-09-12

**Authors:** Andrew Egwar Alunyu, Mercy Rebekah Amiyo, Josephine Nabukenya

**Affiliations:** 1 Faculty of Engineering, Busitema University, Busia, Uganda; 2 Department of Information Systems, Makerere University, Kampala, Uganda; Politecnico di Milano, ITALY

## Abstract

Ignoring the need to contextualise international standards has caused low-resourced countries to implement digital health systems on the ad-hoc, thereby often failing to meet the local needs or scale up. Authors have recommended adapting standards to a country’s context. However, to date, most resources constrained countries like Uganda have not done so, affecting their success in attaining the full benefits of using ICT to support their health systems. They apply the standards ‘as is’ with little regard for their fitness for potential use and ability to fulfil the country’s digital health needs. A design science approach was followed to elicit digital health communication infrastructure (DHCI) requirements and develop the contextual DHCI standards for Uganda. The design science methodology’s design cycle supported DHCI standards’ construction and evaluation activities. Whereas two workgroup sessions were held to craft the standards, three cycles of evaluation and refinement were performed. The final refinement produces the contextualised DHCI standards approved by Uganda’s DH stakeholders through summative evaluation. Results of the summative evaluation show that DH stakeholders agree that the statement of the standards and the requirements specification are suitable to guide DHCI standards implementation in Uganda. Stakeholders agreed that the standards are complete, have the potential to realise DHCI requirements in Uganda, that have been well structured and follow international style for standards, and finally, that the standards are fit to realise their intended use in Uganda. Having been endorsed by DH stakeholders in Uganda’s health system, the standards should be piloted to establish their potency to improve health information exchange and healthcare outcomes. Also, we recommend other low middle income countries (LMICs) with similar challenges to those in Uganda adopt the same set of contextualised DHCI standards.

## Author summary

Most standards are made at international level and often addressed the requirements of countries and organisations that participate in the standards formulation process. Little attention is often paid to address resource constrained requirements of LMICs who are often less represented in these international standards forums. Therefore, these countries find it difficult to adopt and use as is the international standards including standards for digital health communication infrastructure (DHCI). For this reason, Uganda has adapted/contextualised the existing standards to operate within its resource constraints. The main goal for contextualising international standards is to adapt standards to work for the resources constrained contextual environment LMICs. The contextualisation process involved different digital health stakeholders in Uganda who agreed on minimum requirements to implement the standards in Uganda, the standards’ rationale and implementation implication. Furthermore, digital health stakeholders in Uganda evaluated the contextualised DHCI standards and confirmed that that are complete, have potential to realise the country’s digital health requirements, and is fit for purpose.

## Introduction

Studies and strategies in low and middle income countries, particularly in Africa, highlight the need for eHealth standards [1–5]. The digital transformation strategy emphasizes integration, interoperability, and harmonised approaches, emphasizing the importance of standards [6].

Evidence shows that attempts by other countries to standardise their digital health (DH) environment have only involved recommendations of DH standards to be adopted and the development of guidelines for DH implementations/initiatives, such as guidelines for HER, guidelines for protecting the security of health data and privacy for personally identifiable information, etc [1,7–9]. A review of the current standards related to health information exchange (HIE) in Africa revealed three world health resolutions that acknowledge the role of interoperable digital systems in achieving the sustainable development goals (SDGs) [1]. Although Mamunye et al [10] reveal that there is no comprehensive evidence on the current status of HIE policy and standards on the African continent, the African Union [1] identified recommended standards for components relevant to the digital health communication infrastructure (DHCI) such as IEEE 11073-series for interoperable communication among sensor devices and computers, Internet Protocol (IP), Transmission Control Protocol (TCP), User Datagram Protocol (UDP), Transport Layer Security (TLS) Protocol, International Telecommunication Union Telecommunication Standardization Sector (ITU-T) X.509 digital certificates for use in public key encryption, National Institute of Standards and Technology (NIST) Secure Hashing Algorithm 2 (SHA-2), International Organization for Standardization-Technical Standard (ISO/TS) 22600, and ASTM E1985-98. Their endorsement falls short of recommending constituent countries to customise/adapt the standards to respective county needs.

However, there has been calls for countries to contextualise standards [10–13]. This study could not find evidence of low- and middle-income countries (LMICs) like Uganda that have contextualised DH standards. In fact, Mamunye et al [10] recommends that comprehensive, interoperable technical standards be set at each national level and guided by appropriate governance and legal frameworks, data ownership and use agreements, and health data privacy and security guidelines. However, our review of existing works did not find a single framework, theory or practice for DHCI standards that fully applies to Uganda’s local context. Actually, “*there is no one-size-fits-all digital solution for Africa*. *Instead*, *each solution has to be tailored to national and continental needs*, *circumstances and resources*” [1].

These LMICs constrained in resources apply the standards ‘as is’ with little regard for their fitness for potential use and ability to fulfil the country’s digital health needs (specific requirements). Standards with high-end specifications such as (a) bandwidth requirements for DH data sharing may not be achievable in LMICs like Uganda, where there is intermittent internet connectivity and sometimes low data rates in some parts of the country, (b) high-resolution requirements for data capture and presentation, (c) well trained and technical personnel to use available technology to analyses health data, etc. It is, therefore, necessary to customise these standard specifications to the contextual requirements of a health system. Contextualisation involves incorporating local factors and embracing co-production with stakeholders using iterative research cycles [14]. The concept of standards adaption/contextualisation is premised on the argument that international standards might not adequately represent the needs and requirements of a country [13], but require customisation.

Standards for the DHCI cover the scope of ICT hardware devices, computer communication networks, eHealth software systems/application technologies, security and privacy, and eHealth technologies [15]. Therefore, our focus on standards for the DHCI is centred on supporting the interoperability of the eHealth physical infrastructure, networking/ connectivity, human resource factors, and aspects of security and privacy [15–17]. However, a pertinent issue around eHealth interoperability (which remains a significant challenge in LMICs and developed countries) relates to how the diverse hardware and software technologies interface. According to Choi et al [18], compatibility is required to ensure that hardware and software interface seamlessly. In this DH environment, system compatibility is maintained by standardising the electronic communication infrastructure, such as enforcing standards around operating systems, DH applications, communication networks, and computer hardware devices.

The need for DH standards for Uganda was earlier expressed in the MoH’s 2016–2021 strategy [4]. To fulfil Uganda’s national eHealth strategic pillar of eHealth Enterprise Architecture, Interoperability and Standards [4], the study conducted an exploratory study of Uganda’s health system to understand the status of DH standardisation and determine requirements for standardising the DHCI. These activities align with the ITU-T Recommendation I.130, which calls for standard developers to perform a feasibility study before any formal specification work [19]. The requirements for contextualised DHCI standards which were also derived to meet the standards challenges identified from literature and field survey of Uganda’s health system, guided the formulation of contextual DHCI standards for Uganda. The set of DH standards requirements are published on Uganda’s MoH website [20]. DHCI specific requirements are summarised as presented in S1 Table. It should be noted that DH stakeholders have validated the requirements as to what they would like to see realised in Uganda to support HIE. The same Table also presents results of validating requirements for contextualising DHCI standards for Uganda.

Therefore, standards for digital communication infrastructure, such as structured cabling, networking, connectivity, security, and use, need to be adapted to support DH in Uganda’s health system. However, an earlier study of the state of standardisation in Uganda’s DH system revealed that existing DH standards are being applied ad-hoc without (a) customisation and or (b) formal agreement among health stakeholders and all relevant government ministry, department or agencies (MDAs) regards which ones should be used in Uganda [21]. This unauthorised and unregulated application of standards has resulted in DHCI implementations lacking interoperability [4,22], negatively affecting HIE. Such failure to contribute to improved healthcare and elicit better health outcomes have resulted in reported cases of failed digital health interventions in Uganda [22,23]. Which failures can be traced back to standardisation problems that engulf LMICs, including lack of a formal standardisation process, little industry involvement in standards development, inadequate funding for the standardisation process, insufficient human resources, inadequate technical infrastructure for standards participation, competing and overlapping of standards, and complexity of DH data/information and its components [24–27]. Yet standards are needed to foster effective HIE, co-existence and interoperability of systems.

A review of relevant literature showed that for a country to have a set of standards that formally guide its operations, it must either adopt, adapt, or develop itself. Adoption means using the standard as is, i.e., the country accepts adherence to all standards’ requirements. This study chose to use the concept of contextualising standards, i.e., adapting or adjusting to the contextual needs of an environment. Where no relevant standard is available, we recommend the development of the standard. Therefore, the researchers used several standards, network protocols, guidelines, and best practices to inform the framing of contextual standards for DHI to support HIE in Uganda.

Standards contain four key features that ensure clarity, ease of understanding and common interpretation required to support proper implementation [7,28–31]. The features are code and name, statement, rationale/objectives, and implication specification. A standard’s code and name represent an assigned serial number or code and an expression of what the standard is about. Then a standard statement defines and or describes the standard for what it is. The rationale/objective of the standard states presents the basis of reasons for the standard. Implication refers to what needs to be done to implement the standard properly. Therefore, to cater to a resource-constrained environment’s needs, minimum requirements to implement a standard are stated under implication. Finally, the standards’ rationale and implication statement represent the standard’s relevance and applicability in the real-world context.

Therefore, this paper aims to present the contextualised DHCI standards and views of Uganda’s digital health stakeholders regarding its suitability to support HIE in Uganda’s health system. The suitability of DHCI standards is assessed using completeness, the potential to realise the country’s requirements for it, style and structuring, and fitness for purpose. The sole purpose of contextualising is to develop standards that can work for a contextual environment as recommended for African countries [1,13].

## Methods

The study aimed to address HIE challenges in Ugandan government-owned and private health facilities by developing and validating contextualized DHCI standards, involving domain experts and users.

**Participants in the Standards Contextualisation Process–**the researchers engaged domain experts as encouraged by Currie et al [32]. Stakeholder participation in the standards contextualisation process are summarized in Table 1. In order to elicit domain knowledge and insights, participants were drawn from the national standards body, ministry of ICT, telecommunications and regulating body (NITA-U), MoH and digital health researchers because of their diverse roles and interests in organising, regulating and governance of digital health communications in Uganda. Their insights were sought to shape the DICH standards (artefact) that align with Uganda’s health system needs and requirements. First group helped determine candidate standards for contextualisation using the criteria in S3 Table. Then workgroups two and three refined developed and refined DHCI standards to ensure that (a) the standards comprehensively cover all the components of DHCI, (b) they are correct and complete, and (c) they are sufficient to meet the DHCI expectations in Uganda’s health system.

**Table 1:** Tasks and Participants in the DHCI Standards Contectualisation Workgroups.

	Tasks for each DHCI standards workgroup	Participants in each Workgroup	Gender	Education Level
**Workgroup 1**	To select standards for contextualisation from candidate global DHCI standards and best practices	Uganda National Bureau of Standards (UNBS)	Male = 2; Female = 0	Bachelors = 06(60%) Masters = 01(10%) and PhD = 03(30%)
		Ministry of ICT (MoICT)	Male = 0; Female = 1	
		Uganda National Information and Technology Authority (NITA-U)	Male = 1; Female = 0	
		Ministry of Health (MoH)	Male = 2; Female = 1	
		Health Informatics Researchers (HIR)	Male = 2; Female = 1	
**Workgroup 2**	To assess the completeness of the DHCI standards to refine it through rewriting and/or adjusting the name, statement, and requirements specification. Besides the workgroup was tasked to fill in any possible gaps to ensure completeness of the standards in meeting DH requirements in Uganda components of the standards framework to inform refinement.	UNBS	Male = 1; Female = 0	Bachelors = 02(40%) Masters = 01(20%) and PhD = 02(40%)
		NITA-U	Male = 0; Female = 1	
		MoICT	Male = 0; Female = 1	
		MoH	Male = 1; Female = 0	
		Health Informatics Researchers	Male = 1; Female = 1	
**Workgroup 3**	To assess both completeness and potential applicability/ relevance of the contextualised DHCI standards to satisfy DH needs in Uganda. The workgroup focused more on adjusting the specifications of the standards, minimum specifications for hardware components, quality of service to be provided and security measures to protect health data.	UNBS	Male = 1; Female = 0	Bachelors = 09(64.3%) Masters = 05(35.7%) and PhD = 0(0%)
		NITA-U	Male = 1; Female = 0	
		MoICT	Male = 0; Female = 1	
		MoH	Male = 2; Female = 2	
		UCC	Male = 1; Female = 0	
		Implementation Partners	Male = 1 Female = 1	
		Development Partners	Male = 2; Female = 0	
		Health Facility and District-level Users	Male = 1 Female = 1	

The majority of participants in all workgroups were male, with only a few females representing gender placement in the DH employment roles. In terms of highest level of education, stakeholders in the DHCI standards creation were mostly bachelor’s degree holders, with a few master’s and very few PhD holders, reflecting Uganda’s burgeoning health informatics area.

**Standards Contextualisation/Development–**standards determination followed the process that we earlier developed to fast-track standardisation of DH standards in LMICs [16]. The contextual standards are coined from a number of international standards (see subsection on contextualised DHCI standards) that were chosen using criteria in S3 Table.

The study adopted the European Parliament argument that where international standards exist, it is prudent to use them, or the relevant parts, as a basis for the standards being developed, except where such standards or relevant parts would be ineffective [17], then new standards can be created. Therefore, standards selection criteria were used to determine the suitable standards DHCI for adoption or customisation/adaption. An example of how it was used is shown in S3 Table. Where the standards were to be adapted, the scope of contextualisation broadly included;

- Adding local context on top of the recommended standards,
- Developing standards’ statements and rationale to address our implementation context, and
- Stating the standards’ implementation specifications in a manner that addresses Uganda’s limited resources context.

Relevant global standards were not found for the following subcomponents of the DHCI: (a) support by Telephone Companies for DH as part of their social responsibilities, (b) human resource capacity development and training, (c) financial resources, and (d) the provision of sustainable electrical power to support DH systems. Standards were; (a) derived by the researcher, (b) the derived standards were revised/improved by the two workgroups (in Masaka and Makerere), and (c) validated by other DH stakeholders in Uganda. The development process involved;

- Formulating a set of standards for requirements that did not have relevant global standards or for which suitable best practices were not found elsewhere, and
- Defining implementation specifications for DHCI practices suited for Uganda’s health system context,
- Seeking agreement among workgroup members regarding the standards’ statement, rationale and specifications.
- Documenting of standards based on agreed-upon structure, i.e., standard code, name, statement, rationale, and implementation specification.

**Validation of the Contextualised DHCI Standards**–DHCI standards refinement workgroups consisted of five participants who refined the contextualised standards in two rounds. During this, they critically reviewed, rephrased, and adjustment the name, statement, and standards implementation specifications. Where they could not agree on improvements, the standards workgroup recommended that the researcher conduct further consultations and literature reviews to improve the standards. A third and final summative evaluation was done by seventeen participants who included DH users at the health facility, a District health officer (DHO), a district Biostatistician, standardisation officers from NITA-U, MoICT, UNBS, and MoH, implementing partners, and health informatics researchers. They assessed the completeness of the standards, requirements to be realised by the standard, style and structure in which the standards have been framed, and fitness of the contextualised standards for guiding DHCI implementations in (potential use) Uganda.

**Approval Process**–Stakeholder-approved standards were submitted to the MoH as the problem owners in Uganda. Members of the ministry’s technical working group have approved the standards. The same was presented to senior management, who approved it. The MoH, working in collaboration with PATH and CDC in Uganda, received expert feedback on DH standards as a national technical document that they were required to review. Concerns concerning DHCI standards included (a) recommendation for using WPA2 or WPA3 instead of WEP as a security feature and (b) that clear reference/statement be made on data backup and steps towards data recovery. These concerns were addressed; therefore, the standards await approval by top management as a national document that will guide DHCI implementations in Uganda’s health system.

**Ethical Consideration**–Engagement of DH stakeholders did not pose potential risks to patients’ personally identifiable data or infringement to Uganda’s data protection policy [33] and so, Makerere School of Public Health’s Institutional Review Board granted ethical approval for the study to be conducted in Uganda. Furthermore, Uganda’s Ministry of Health provided permission to engage DH stakeholders in Uganda.

## Results

### Demographics of participants in the standards evaluation process

**Fig 1:**
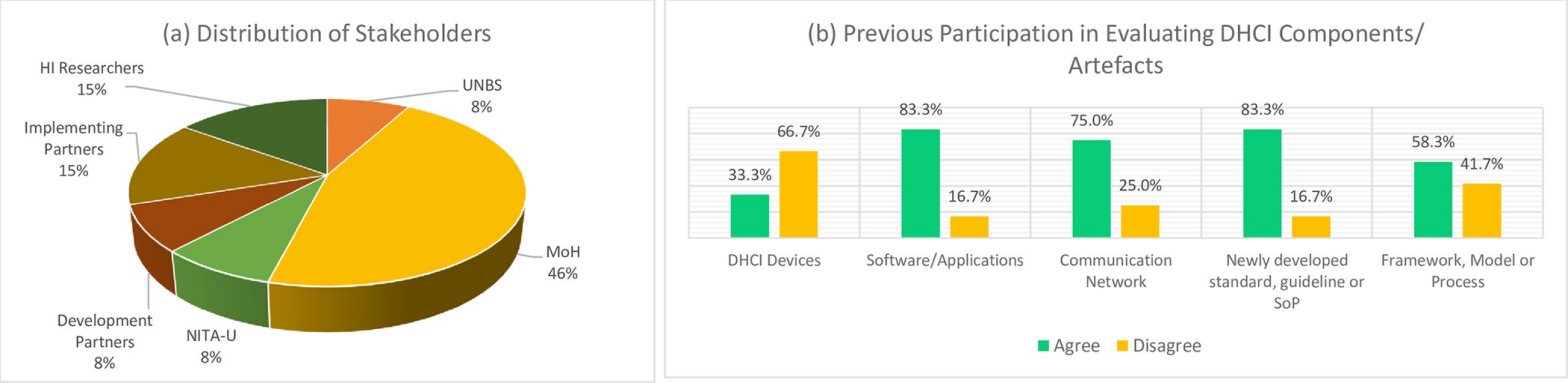
Distribution of Stakeholders who Validated Contextualised DHCI standards and the standardisation.

Fig 1 shows the distribution of stakeholders who validated the developed DHCI artefacts. Fig 1(A) shows that most respondents (46%) were MoH officials by stakeholder category, especially drawn from the health informatics division. 15% of the respondents were drawn from the implementing partner category, and another 15% from Health informatics researchers in Uganda. Finally, UNBS, NITA-U, and Development Partners were represented by 8% each. On their academic qualification, results show that 33.3% and 66.6% have a minimum of bachelor’s and master’s degrees in ICT or health informatics, respectively.

Furthermore, the participants indicate that they have previous experience in the evaluation/validation of IS artefacts. Fig 1(B) shows that out of the 13 participants, 41.7% had previously evaluated DHCI devices; 91.7%, software/applications; 75%, communication network; 83.3% standards/SoPs; and 58.3% in the validation of framework, model or process.

### Contextualised/developed standards

Table 2 shows the contextualised/developed standards for the DHCI required to support HIV/TB HIE for a patient-centred care in Uganda (see S2 Table for details). The contextualised standards for Uganda are informed by international standards for computing computer hardware and telecommunications, networking, software applications, security and privacy, and electrical wiring in healthcare environments needed to address challenges to DHCI in the country’s health system.

**Table 2:** Summary of Source Standards, Contextualised DHCI Standards–Codes, Names and Statement.

Components of DHCI	Source standards/ Best Practice	Contextualised standards		
		Code	Name	Statement of the standard
**DH Hardware Devices**	ANSI/BICSI & Institute, 2018; ISO/IEEE, n.d.; ISO/TR, 2009; TR-42.1, 2017; MoH’s ICT Device Specification; NITA-U, 2013; Singapore Ministry of Health, 2015; The Open Group, 2018); ANSI/TIA-1179 –A; and TIA-942	UG_DHS_CI01_01	Align DHCI Standard Implementations	Critical areas and or changes in the DHCI are appropriately aligned to the country’s digital health needs, eHealth strategy, and related policies
		UG_DHS_CI01_02	DHCI lie within the country’s DH technology blueprint	Minimum specifications of HW devices restrict implementations of the DHCI within the country’s DH technology blueprint that assures healthcare facilities/ organisations participating in the connected health system establish a DH communication network with quality devices, data rate, and network services
		UG_DHS_CI01_03	Minimise diverse implementation of the DHCI	Reduce diversity of DHCI implementations that support healthcare programmes and interventions through procurement and implementation based on contextualised global standards and min. specifications adopted/laid down by MoH and related MDAs
**Communication Networks & Connectivity**	Australian Commission on Safety, 2011; European Parliament, n.d.; European Union, 2015; HIPAA, 2013; ITU-T, 2015; MoH’s Device Specification, work in progress; The Open Group, 2018; TR-42.1, 2017, p.	UG_DHS_CI01_04	Standardised Connected Health Systems	Healthcare facilities/ organisations participating in the connected health system should establish DH communication network(s) with quality devices, data rate, & suitable network services to support secure HIE.
		UG_DHS_CI02_01	MoU Specifying Telecom and Internet service Provision	Any form of MoU and SLA (as part of the social corporate responsibility) between with telecom and Internet service providers should specify both quality and terms of service provision to Uganda’s health sector.
		UG_DHS_CI02_02	Last Mile Connectivity	NITA-U should connect all health facilities to the National Backbone Infrastructure.
**DH Applications, Software and Technologies**	European Union, 2015; Singapore Ministry of Health, 2015; The Open Group, 2018	UG_DHA_IM03_01	Application Design & Development	DH application designing and development is guided by agreed standards and guidelines as contextualised by MoH and related MDAs, which specify modes for linking (sharing) health data between applications in a secure manner.
		UG_DHA_IM03_02	Mandate to Oversee all DHCI Implementation	MoH superintends the implementation and use of DH applications in public and private healthcare sites.
		UG_DHA_IM03_03	Up-to-date and Interoperable Software	Both system and general application softwares that are run on DH devices have update security measures.
		UG_DHA_IM03_04	Acceptable DH Technologies	Control choice of DH technologies to be adopted and used by healthcare organisations, facilities and researchers to ensure interoperable and secure collection, sharing and use of health data/information.
**Security, Privacy and Secure Use of the DHCI**	HHS Office of Civil Rights, 2013; Health IT, 2015; HIPAA, 2013; European Union, 2019; ITI Planning Committee, 2015; EHRS FM R2; ISO/HL710781; ISO IS 17090–1 2013; ISO IS 17090–2 2008; MoICT-Uganda, 2019; The Open Group, 2018	UG_DHS_SP02_01	DH Information Security Access Rights	DH information security access rights are properly assigned and managed
		UG_DHS_SP02_02	DH Data/Information Transmission Security	Develop policy-based guidelines for securing the DH data/information transmission.
		UG_DHS_SP02_03	Data Protection–Confidentiality and Integrity.	Enforce measures that provide for data/information confidentiality and protection are implemented and enforced across the entire health sector.
		UG_DHS_SP02_04	Improve user observance of security and privacy guidelines	Users are conversant and comply with DHCI relevant security and privacy laws, and guidelines including Uganda’s Data Protection and Privacy Act regarding electronic data collection, processing, retention, and exchange.
		UG_DHS_SP02_05	Mitigation of security risks	DH systems have mitigation plans addressing security risks faced by DHCI and the health data they contain.
**Facilitating Resources**		UG_DHC_IT01_01	Training of DH Users	The MoH, collaborating medical training institutions and the DH implementing partners should aim at producing an all-rounded, knowledgeable and skilled digital health workforce capable of observing stipulated guidelines.
		UG_DHC_IT02_01	Training of Managers responsible for DHCI	Leaders/managers responsible for DHCI are informed and trained on the use of DH technologies and trends.
		UG_ DHS_CI03_01	DHCI Financing	Adequate funds should be allocated to finance the DHCI that supports DH data collection, processing and information sharing in Uganda.
		UG_DHS_CI04_01	Reliable Electric Power Supply	Reliable and alternative sources of electric energy are available at all healthcare service delivery points.

The scope of contextualised and or developed standards includes;

1. *Standards and specifications for DH hardware devices*: were informed by ANSI/BICSI & Institute, 2018; ISO/IEC, 2020; NITA-U, 2013; Singapore Ministry of Health, 2015; The Open Group, 2018a; and TR-42.1, 2017 among other standards and best practices around the world that relate to computer hardware devices that can be used in health. The contextual forms of the standards should ensure that specifications of HW devices restrict implementations of the DHCI within the country’s DH technology blueprint ensuring implementers establish systems that support timely, reliable and secure HIE. The specifications for hardware devices are aimed at ensuring that the hardware devices are capable of supporting all forms of approved DH applications, and the reliable capture, processing, presentation, and or sharing of health data and information.
2. *Standards for the DH communication networks and connectivity* were informed by ANSI/BICSI & Institute, 2018; Australian Commission on Safety, 2011; Council of the European Union, 2004; European Union, 2015; HIPAA, 2013; ITU-T, 2015; The Open Group, 2018a; and TR-42.1, 2017 among others. The contextualised standards advocate for establishing a connected DH communication network with quality devices, data rate, and suitable network services to support secure HIV/TB HIE in Uganda trough ensuring that all participating health organisations/ facilities implement DHCI that meet the required hardware, software, network, and security specifications for quality health data. It recommends the adoption of ANSI/TIA-1179-A. Furthermore, the standard stipulates minimum specifications for connectivity devices such as network routers, switches, access points, server machines, and firewalls. Lastly, the standards also stipulate that quality and terms of service provision by telecom and internet services providers supporting healthcare in Uganda.
3. *Standards for DH applications*, *software*, *and technologies aim to ensure that DH applications’ design*, *development*, *and deployment are* guided by standards that specify modes for linking (sharing) health data from across DH applications securely. Suggested standards include US ISO IEC 15489 for records management, US ISO IEC 9075–2011 and later series for databases languages, etc. Furthermore, it provides guides that MoH superintends implementation and use of DH applications in public and private healthcare sites through directing development or acquisition and implementation or rigorous process of testing application softwares and continuous monitoring of use. The standards also direct MoH to be in charge of DH technologies adopted by healthcare organisations, facilities, and researchers to ensure the interoperable and secure collection, sharing, and use of health data/information. MoH is responsible for publishing an atlas (database) of approved ICT technologies that can be used in the country’s health care system.
4. *Standards for the security*, *privacy*, *and secure use of the DHCI* were informed by several international/global standards, best practices and Uganda’s data protection and privacy Act. This standard ensures that DH information security access rights are appropriately assigned and managed, operates security policy guidelines for securing the DH data/information transmission channels, enforces data/information confidentiality and protects personally identifiable data that the DHCI contains, ensures that DH users are conversant and comply with relevant security and privacy laws/guidelines, and finally that all entities in the health sector must mitigate, to the extent practicable, any harmful effect that may result from the violation of security policies and guidelines or requirements.
5. *Standards for the DHCI’s facilitating resources* provide specifications regarding requisite technical and skilled human resources training to perform DHCI tasks while ensuring the security of the systems and data that they contain. It also stipulates the need to identify, solicit and distribute funds for procurement, implementation, maintenance/updating and replacement of the DHCI components. Finally, the standards stipulate what needs to be done to ensure. Safe, reliable, and alternative electric energy sources are available at all healthcare service delivery points.

### Validation results

To obtain participants’ views regarding the completeness of the contextualised DHCI Standards, they were assessed for completeness, DHCI requirements to be realised by each standard, style and structuring of the standards, and fitness of the standards for potential use in Uganda’s health system.

#### (a) Completeness of DHCI standard for Uganda

**Fig 2:**
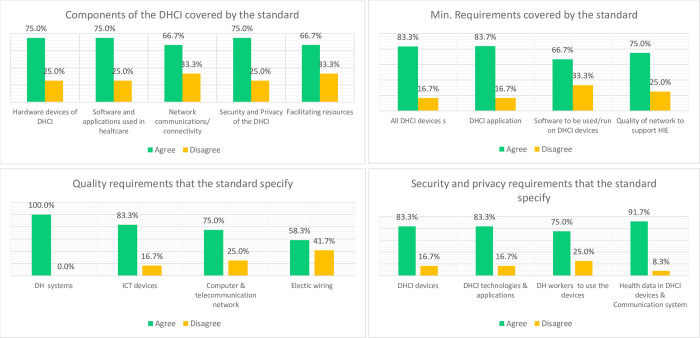
Participants’ views regarding Completeness of the Contextualised DHCI Standards.

Results in Fig 2 show respondents were satisfied with DHCI standards coverage regarding scope, specification minimum requirements, quality, and security for all the DHCI components. The lowest record of the agreement was on the quality of electrical wiring and network cabling at 58.3% each. There were higher percentages of agreement of 100% on requirements for DH systems, 91.7% on the security of health data that the DHCI devices and communication system carries, 83.3% on minimum requirements for DHCI devices and applications, quality requirements for the ICT devices, and security requirements for DHCI devices, technologies and applications. However, the rest still registered majority agreement on all metrics used to assess the completeness of the contextualised standards for DHCI for Uganda. All the respondents agreed that the standards provide complete coverage of the DHCI components. In addition, one respondent highlighted that the standards for “*the DHCI goes further to elaborate minimum requirements to procure DHCI equipment and governing laws and frameworks*” (VR09, Health Information System Analyst). Meaning they were satisfied with the details provided by the standards.

#### (b). Potential to realise DHCI standards requirements

Table 3 shows responses regards DHCI requirements for DH hardware devices, networks and connectivity.

**Table 3:** The potential of the DHCI standards for hardware devices and communication networks to realise requirements.

**DH Devices**	**Agree**	**Not Sure**	**Disagree**
Stipulate min. specifications for DHCI devices to be acquired by participating entities	100.0%	0.0%	0.0%
Recommend to implementers critical areas of the DHCI that need to be funded	91.7%	8.3%	0.0%
Stipulate acceptable forms of electronic health data backup/storage	91.7%	8.3%	0.0%
**DH Network and Connectivity**	**Agree**	**Not Sure**	**Disagree**
Guidelines for the establishment a reliable communication network, both LAN & Internet	75.0%	25.0%	0.0%
Guidelines specify a set of administrative, technical and managerial actions during the life-cycle of DH comm. Network	83.3%	16.7%	0.0%
Specify expected QoS to support all forms of DH data exchange: Query-based, consumer-mediated, & managerial-based exchanges	83.3%	16.7%	0.0%
Specify expected QoS to support reliability & high data rate/bandwidth	75.0%	16.7%	8.3%
Specify the contents of an MoU with Internet providers	83.3%	8.3%	0.0%
State min. specifications to be followed when implementing a DH communication network	83.3%	16.7%	0.0%

***DH Hardware Devices***: All participants agreed that standards for DH hardware stipulate minimum specifications for all hardware devices to be implemented by participating entities. Besides, 91.7% of the participants agreed that the standards for DH hardware devices recommend critical areas to be funded. Another 91.7% of the participants agreed that DH hardware device standards specify DH data backup/storage forms. Overall, no single participant disagreed with any of the three statements about standards for the DHCI hardware devices.

***Connectivity and Communications Network***: Concerning the DH data communication network, the majority, 83.3% of the participants agreed that the standards (1) state minimum specifications be followed when implementing a DH communication network; (2) specify contents of any MoU with Internet providers, (3) specify expected QoS to support all forms of DH data exchange, and (4) provide guidelines for administrative, technical and managerial actions during the life-cycle of the DH communication network. In addition, 75% of the participants agreed that the standards specify expected QoS to support reliability and high data rate/bandwidth for DH data sharing. Another 75% also agreed that the standards provide guidelines for establishing a reliable communication network, both LAN and the Internet required to support DH data communication.

**Fig 3:**
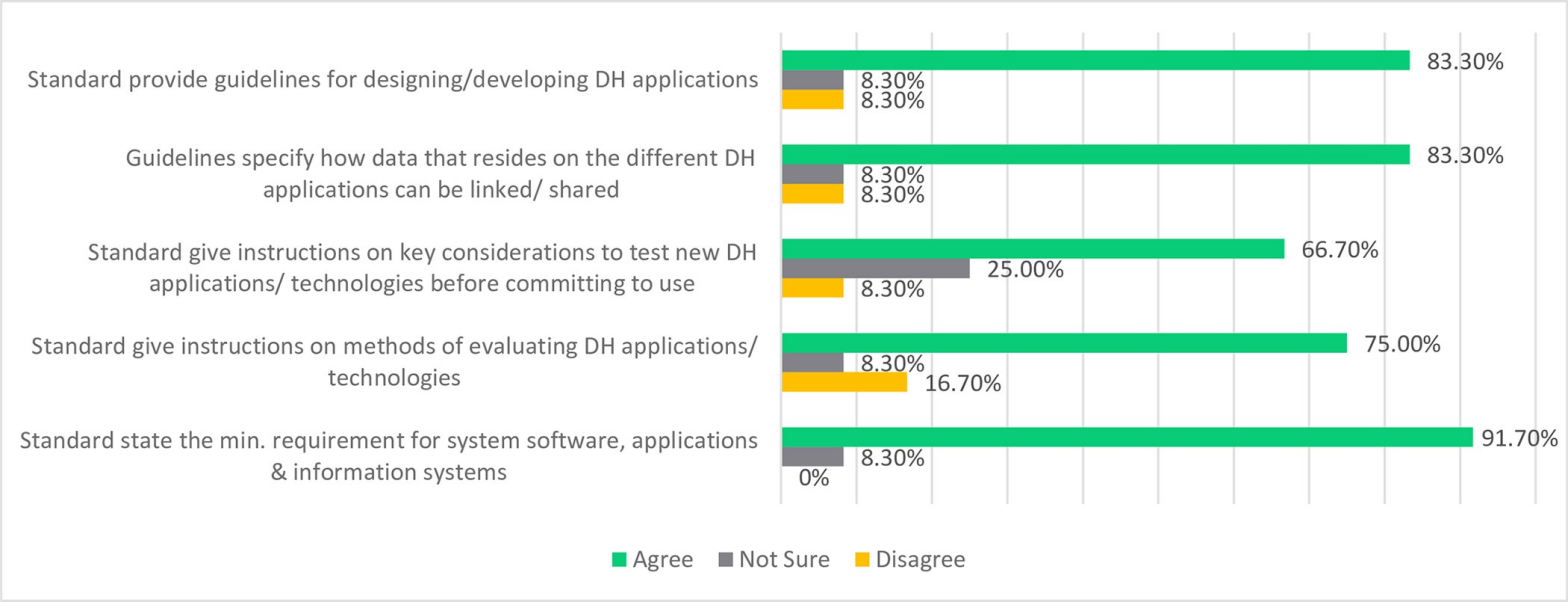
Participants’ views regarding Contextualised Standards for DHCI Application and System Software.

***DH Applications and Software***: To support interoperability and system integration, 91.7% of the participants agreed that the standards state minimum requirements for the DHCI software, applications, and information systems (see Fig 3). 83.3% of the participants agreed that the standards provide guidelines (1) for designing/developing DH applications and Information Systems and (2) that specify how data that resides on the different DH applications/information systems can be linked or shared across applications.75% agreed that the standards give instructions on methods for evaluating DH applications/technologies. However, 16.7% disagreed. Only 66.7% of the participants agreed that the standards give instructions on critical considerations for testing new DH applications technologies before committing to use. In this case, 25% of the participants remained neutral, signifying a significant number were uncertain about the requirements for testing new DH applications. In all other instances, 8.3% of the participants remained neutral.

***Security*, *Privacy and Use of DHCI*:** Several standards exist for all dimensions of DHCI. Table 4 shows that all the respondents (100%) agreed on two of the security and privacy statements, i.e., the standards outline how the cardinal principles of confidentiality, integrity, and availability (CIA) should guide policies/controls are designed to protect health data and that standard instruct on how personally identifiable data should be protected. Furthermore, 91.7% of the respondents agreed that the standard (a) aligned with the national Data Protection and Privacy Act and the international information security standards, (b) Standard provides for all forms of safeguards against security threats and vulnerabilities, i.e., administrative, technical and physical security, (c) provide recommendations for organisational requirements for protecting the DHCI and data they contain, (d) provide recommendations for policies, procedures & documentation requirements for securing the DHCI and data they contain, and (e) cover physical security, health information/data protection, DH assets, potential threats, vulnerabilities and impacts. Finally, 83.3% of the respondents agreed that (a) the standard instruct how risks faced by health data residing on DH systems can be mitigated and (b) security and privacy guidelines tailored to Uganda’s DH policy/strategy. Although a few respondents (8.3%) disagreed on two of the nine metrics used to measure the contextualised security and privacy standards, some remained neutral regards six of the nine metrics.

**Table 4:** Responses regarding Contextualised Standards for Security, Privacy and Use of the DHCI.

Security and privacy requirements to be realised by the standard	Agree	Not Sure	Disagree
Security and privacy guidelines are tailored to Uganda’s DH policy/strategy	83.3%	16.7%	0.0%
Security and privacy guidelines are aligned with national & international information security standards	91.7%	8.3%	0.0%
Standard outlines how cardinal principles of CIA should guide policies/controls to protect health data	100%	0.0%	0.0%
Security and privacy standard instruct on the protection of personally identifiable data	100%	0.0%	0.0%
Standard provides for all forms of safeguards against security threats and vulnerabilities	91.7%	0.0%	8.3%
Standard provides instructions for physical safeguards against security threats & vulnerabilities	91.7%	0.0%	8.3%
Standard provides instructions for technical safeguards against security threats and vulnerabilities	91.7%	0.0%	8.3%
Standard recommends organisational requirements for protecting the DHCI & data they contain	91.7%	8.3%	0.0%
Standard recommends policies, procedures & documentation requirements for securing the DHCI & data they contain	91.7%	8.3%	0.0%
The scope of security standards covers all security dimensions	91.7%	8.3%	0.0%
Standard instruct how risks faced by DH health data can be mitigated	83.3%	8.3%	8.3%

***Facilitating resources (Personnel Capacity*, *financial and Electric power)*:** Table 5 show responses regards standards for DHCI facilitating resources. Responses on standards show that 100% of the respondents agreed that the standard (a) specifies the type and scope of the DH training suitable for healthcare professionals that use the DH systems, (b) recommends DH curricular revisions that must be made to produce digitally empowered healthcare professionals and (c) guide on the equitable allocation of minimal resources to critical areas of the DHCI to ensure a functional, connected health system even amid resources constraints.91.7% of the respondents agreed that the standard provides guidelines for the use of clean /renewable energy such as solar, wind and biogas, among others to power the DHCI devices, communication network and systems. In addition, 83.3% of the respondents agreed that the standards (a) specify roles/responsibilities of users, administrators, and managers of the DHCI, (b) recommend upgrades or in-service training to be provided to the current DH workers to make them digitally enabled, (c) guide the MoH and implementing entities on how to identify possible sources of funding that can be used to support procurement, deployment, and maintenance of the DHCI devices, communication networks, applications, enforce adherence to security and privacy measures and even facilitate training of the human resources which will use the systems. Finally, 75% of the respondents agreed that the standard for facilitating resources (a) specifies the minimum skills/knowledge set required of a DH worker to use the DHCI adequately and (b) specifies requirements for sustainable electric energy to power all the DHCI devices and communication systems.

**Table 5:** Responses regarding Contextualised Standards for DHCI Facilitating Resources.

Facilitating resource requirements to be realised by the standard	Agree	Not Sure	Disagree
State roles/ responsibilities of users, administrators, and managers of the DHCI	83.3%	16.7%	0.0%
Standard specifies the type & scope of DH training suitable for healthcare professionals	100%	0.0%	0.0%
Specifies of min. skill/knowledge set for DH workers who use the DHCI	75.0%	16.7%	8.3%
Standard recommends upgrades or in-service training of DH Workers	83.3%	16.7%	0.0%
Standard recommends DH curricular /training programme revisions	100.0%	0.0%	0.0%
Standard instructs MoH/implementing entities to identify possible sources of funding	83.3%	16.7%	0.0%
Standards guide the equitable allocation of the minimal resources to critical areas of the DHCI	100%	0.0%	0.0%
Standards specify requirements for sustainable electric energy to power all DHCI devices and systems	75.0%	25.0%	0.0%
Standard guides on the use of clean energy (renewable energy)	91.7%	8.3%	0.0%

#### (c). Style and Structure of the DHCI Standards

**Fig 4:**
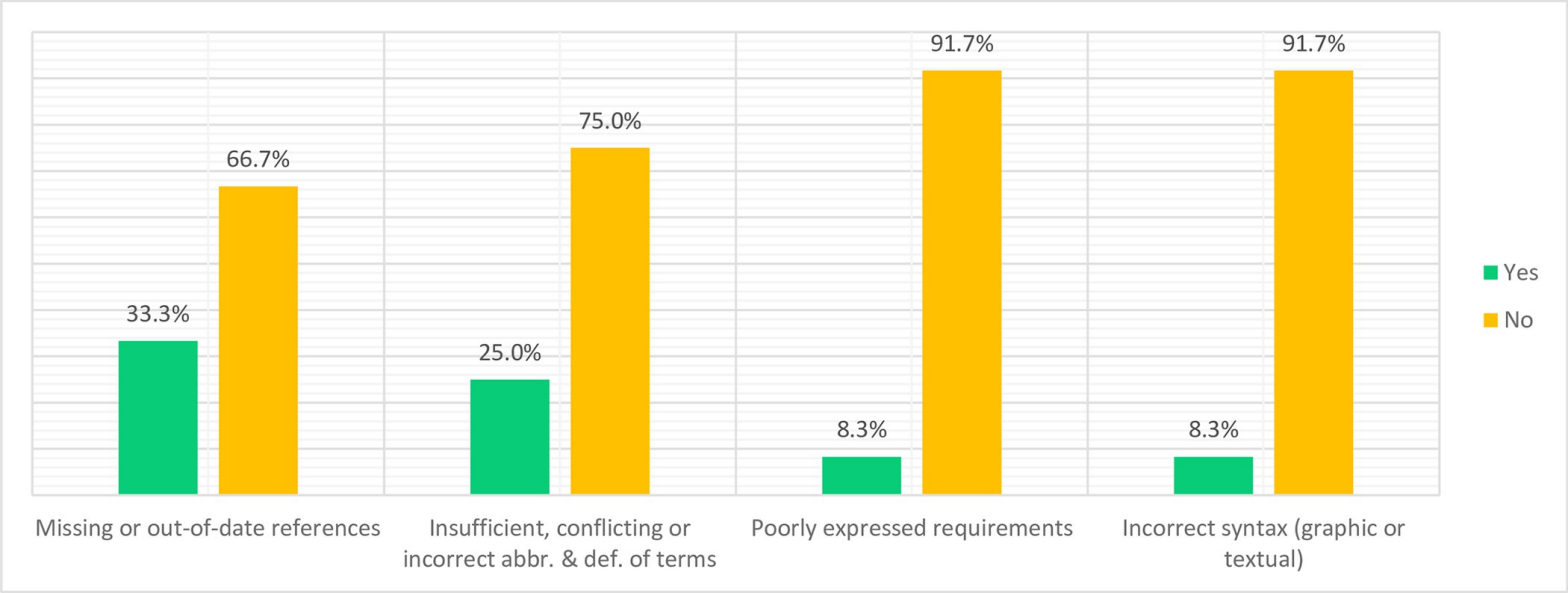
Participants’ views regarding Style and Structuring of the Contextualised Standards for DHCI.

The scope of style and structuring of the standards included compliance with standard writing principles and editorial correctness. When asked to identify any other editorial issues that may negatively impact the understanding and use of the standards, there were mixed reactions; therefore, no majority of participants could agree on any editorial issue. In fact, most participants disagreed on all editorial standards issues (see Fig 4). For example, although 33.3% of the respondents thought that some of the references were missing, 25% thought there were insufficient, incorrect or conflicting definitions/abbreviations. Only 8.3% believed that the standards were poorly expressed and had problems with syntax. The majority thought the standards were well structured and followed the proper style.

Respondents agreed with the style and structure with which DHCI standards were developed. Overall, participants (a) showed satisfaction with the clarity and ease with which the standards can be read and understood. For example, one participant said, “*the requirements are easy to read and understand*” (VR01, Information Systems Consultant).

Another argued that the standards are “*well specified and clear*” (VR03, Information Technology Officer), yet another agreed that “*The standard is easy to read and understand by all users across the of the healthcare system*” (VR09, Health Information System Analyst) (b) Consented that the standards are clearly expressed and easy to understand, they agreed that the contents are properly expressed in such a way that they can guide monitoring of implementations of the set standards (VR05, Data Scientist; VR07, Information Systems Consultant; VR08, DH Technical Advisor; VR09, Health Information System Analyst; VR10, DH Program Coordinator; VR11, Data Governance and Security Specialist; VR12, Information System Supports Officer). (c) Agreed that the references are accurate, wider-ranging, and focused on the individual components of the DHCI (VR05, Data Scientist; VR07, Information Systems Consultant; VR08, DH Technical Advisor; VR09, Health Information System Analyst; VR10, DH Program Coordinator; VR11, Data Governance and Security Specialist; VR12, Information System Supports Officer). Although some participants were not certain of the reference to other standards, those with possible experience in the guideline/standards development process confirmed that the references exist and are correct. For example, a respondent, VR09 (Health Information System Analyst) claimed “*references to the existing standards are accurate*”, and VR10 (a DH Program Coordinator) confirmed that “*they are very well referenced to other standards*, *especially international ones*”.

#### (d). Participants’ views on Fitness of the Contextualised DHCI Standards for Intended Use in Uganda

Among other things, technical and editorial issues influence a standard’s fitness for the intended use. It’s an assessment of the efficacy of the standard, i.e., the degree to which the standard will produce its desired effect. Uganda’s ministry of health desires to have a connected health system where all DH systems are interoperable, providing improved efficiency, healthcare decision-making, better procurement process, and overall cost reduction in healthcare management. Therefore, we assessed (a) DH practices affected by DHCI standards’ implementation and (b) the potential benefits of implementing DHCI standards.

**Fig 5:**
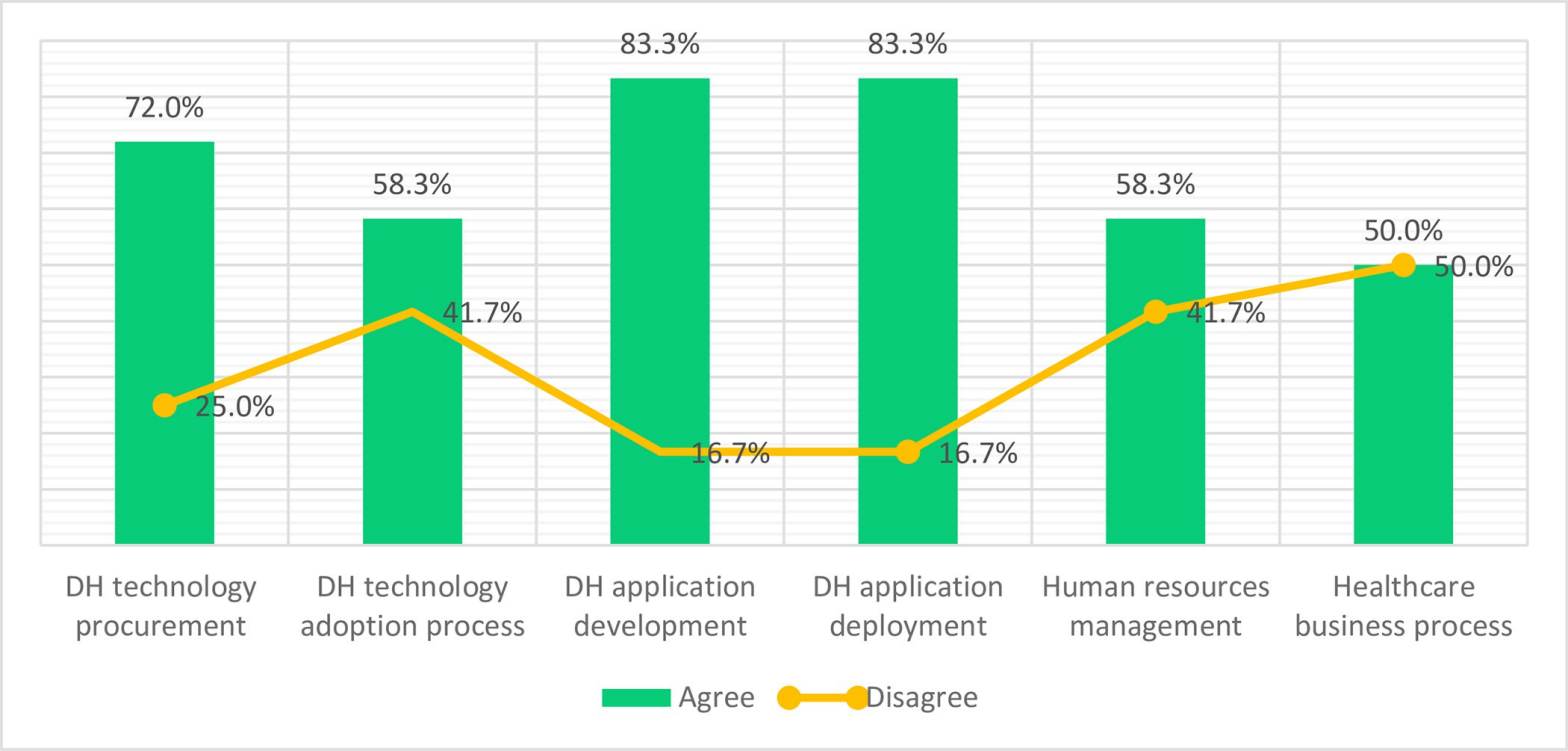
Digital Health Practices Affected by Application of Contextualised DHCI Standards.

***Participants’ views regarding DH Practices that could be affected by DHCI standards Implementation***: Respondents were presented with seven DHCI implementation practices/processes and asked which of them were affected by the decision to apply the standard (see Fig 5). Respondents identified several practices affected by the decision to apply contextualised standards for the DHCI. Whereas 83.3% of the respondents agreed that implementation would affect DH application development and deployment, 16.7% disagreed. Though 75% of the respondents agreed that the application of the standard would influence the DHCI procurement process, 25% disagreed. Although 58.3% of the respondents agreed that the standard would affect the DH technology adoption process and human resources recruitment, 41.7% disagreed. Finally, there was a split response of 50% on whether the standards will affect the healthcare business process.

**Fig 6:**
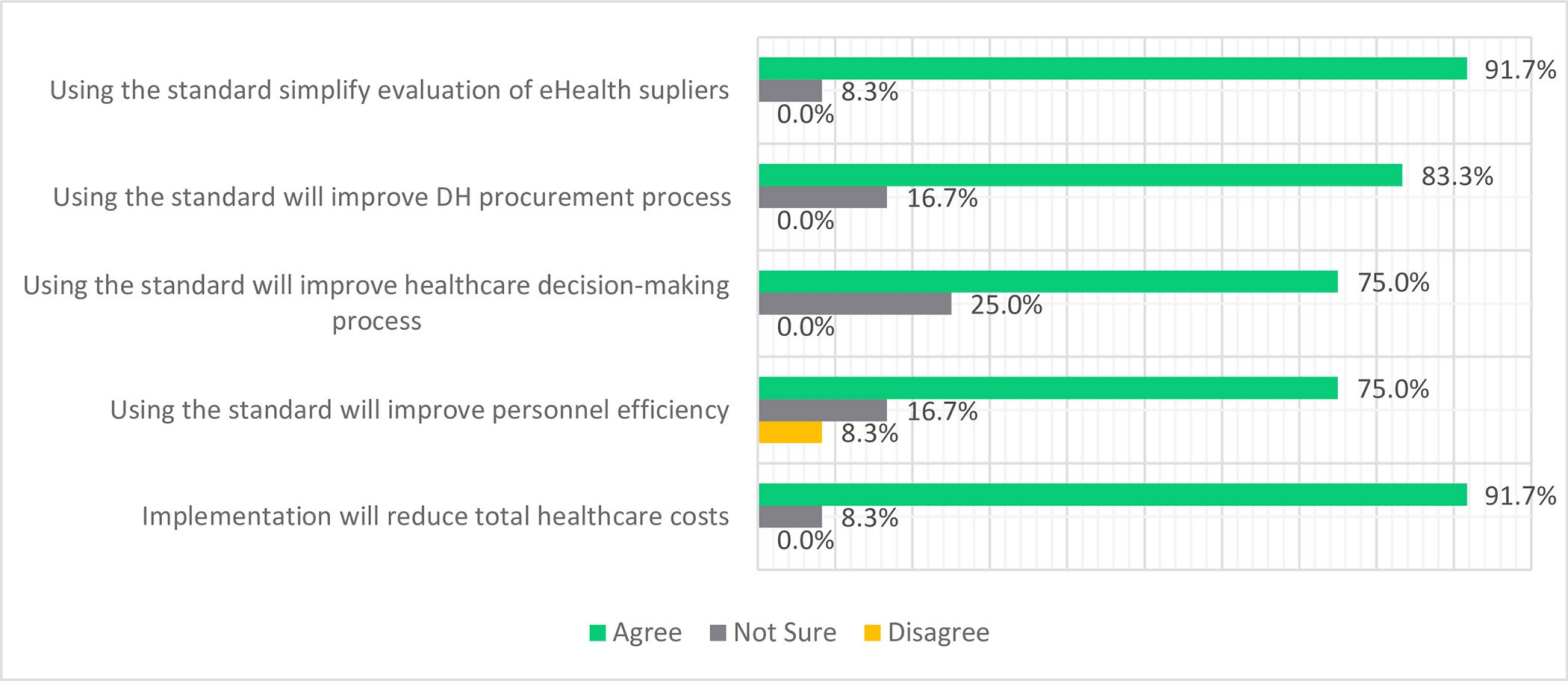
Potential Benefits of Implementing DHCI Standards.

**Potential Benefits of Implementing DHCI Standards**: Results in Fig 6 show that 97.1% of the participants agreed that applying the standard will improve HIE, simplify the evaluation of DHCI supplies, and reduce total healthcare costs. 83.3% of the participants agreed to improve the DHCI procurement process, and 75% of the respondents agreed that the standards would improve healthcare decision-making and personnel efficiency.

However, 25% of the students were unsure whether implementing the standards would improve healthcare decision-making. Another 16.7% of the respondents were not sure of the potential benefits of the standard to improve personnel efficiency or the procurement process. Only 8.3% of the respondents remain uncertain about the potential of the standards to simplify the evaluation of the DHCI supplies and reduce overall healthcare costs. The only disagreement was registered at 8.35 for improved personnel efficiency.

## Discussion

Contextualised standards for the DHCI were assessed for completeness, the standard’s requirements to be realised, its style and structure, and fitness for potential use.

**DH Hardware Devices:** DH hardware devices are computer hardware used for processing, storing, retrieving, sharing, and using healthcare information for communication and decision-making [34]. These devices require minimal specifications to meet healthcare reliability and time constraints. Standards for DH hardware devices aim to guide quality procurement, minimize diverse implementations, and align them with the country’s digital health needs, ensuring efficient patient care and health decision-making.

**DH Communication Network and Connectivity:** Nearly all DH applications demand enhanced quality of service (QoS), security, and broadband technologies, i.e., high bandwidth, low latency, always available, security/privacy, and ubiquity [35]. ANSI/TIA-1179 Healthcare Facility, Telecommunications Infrastructure Standard, recommends that healthcare networks should support high Gb/s speeds [36]. However, broadband access is usually unavailable in LMICs, especially in rural areas [37]. These demands for enhanced QoS imply the infrastructure be standardised across healthcare facilities and or organisations for better HIE; otherwise, one end would fail the sharing [38]. Furthermore, Martinez et al [37] suggested an analysis of available resources to improve the management of the QoS, which may require implementing a minimum resource specification. The minimum communication requirements that guarantee QoS differ by setting, as exemplified in the statement: “*the connectivity needs of healthcare facilities are far more complex than commercial buildings and can be optimised only through a standard that recognises their particular and often unique requirements*” [39,40]. The contextualised DHCI standards for networks and connectivity address concerns from the field study of Uganda’s health system and therefore cater for such resource requirements. South African National Department of Health [41] argue that the standard should be specific about the nature and expected QoS. DHCI standards communication stipulate the need for specifying QoS, among other things, thereby providing a common reference to technology and design practice, networking and cabling. However, success depends on planning, designing, implementing, and managing such a network following stipulated standards. The planning and design of a communication infrastructure require up-to-date information on user needs, best-current practices, and projection of the facility’s future needs [40].

Healthcare facilities (DH) applications require enhanced quality of service (QoS), security, and broadband technologies, i.e., high bandwidth, low latency, always available, security/privacy, and ubiquity [35]. The ANSI/TIA-1179 Healthcare Facility Telecommunications Infrastructure Standard recommends high Gb/s speeds for healthcare networks [36], but broadband access is often unavailable in low- and middle-income countries (LMICs), particularly in rural areas [37]. Standardization across healthcare facilities and organizations is crucial for better HIE, otherwise, one end would fail the sharing [38]. Analyzing available resources can improve QoS management, potentially requiring minimum resource specifications [37]. The minimum communication requirements that guarantee QoS differ by setting, as exemplified in the statement: “*the connectivity needs of healthcare facilities are far more complex than commercial buildings and can be optimised only through a standard that recognises their particular and often unique requirements*” [39,40]. The South African National Department of Health [41] advocates for specific QoS requirements in DHCI standards. Success depends on planning, designing, implementing, and managing a network following these standards, which require up-to-date information on user needs, best practices, and projections of future facility needs [40].

**DH Applications, Software, and Technologies:** Many DH applications in low-middle income countries (LMICs), including Uganda, lack interoperability [42–44]. Efforts to solve this problem have not been successful, and countries are pushing for solutions. The diversity of software and technologies without adherence to common standards is a hindrance to interoperability [23]. To address this, a DH enterprise and standards approach is proposed [4,45]. This approach provides contextual standards for DH applications, software, and technologies to support HIE in Uganda. Stakeholders agree that these standards provide guidelines for DH innovations and interventions (see Fig 3), reducing diversity and improving interoperability.

**Security and Privacy:** This work focuses on the security and privacy of DHCI hardware devices, communication networks, health data, and secure use/handling of these components [46]. Despite infrastructure components, software, and technology systems providing security measures, security and privacy fears hinder the widespread adoption of DH technologies [47]. The security and privacy of health data held by DH systems are linked to hardware and software security [48]. The contextualized DHCI standards cover all domains of DH security.

**Facilitating Resources:** The DHCI facilitating resources include human resources, funding, and electric power. Common DH competency domains include basic information technology literacy, health information management, eHealth data interpretation and analysis, digital communication, ethical requirements, and digital data security [49–52]. These competencies are covered in Ugandan standards for training DH users and managers. Financial limitations have been a major reason for DH interventions failure in LMICs. Contextualized standards guide MoH and implementing entities in developing sustainable funding plans for DH initiatives. Reliable electric power is essential for Intensive Care Unit (ICU) environments [53]. It is important to continue discussing the optimal use of the limited resources in LMICs as part of the sustainability plan [54]. Finally, it is impossible to progress DH investments without financial, competent human and ICT infrastructural resources [55].

The standard for DHCI in Uganda has been validated by stakeholders, demonstrating its consistency, clear terminologies, and non-conflicting requirements. The standards are expected to improve the evaluation of suppliers, procurement processes, personnel efficiency, healthcare decision-making, and overall healthcare costs. Validators from all DH stakeholder groups in Uganda agree that the standards could be adapted for use within the country’s healthcare system. The standards will guide the procurement, implementation, and use of DHCI hardware devices, establish communications networking for health data requirements, determine digital health applications, software, and technologies, set security mechanisms, and determine resources required for standardization and implementation. These standards align with high-quality standards [56] and aim to streamline the procurement process, improve healthcare decision-making, and reduce total healthcare costs.

## Conclusion and recommendation

The study set out to develop contextual standards to support deploying a digital communication infrastructure capable of facilitating HIE in Uganda’s health system. The standards were grounded on existing international standards and best practices for components that comprise the communication infrastructure. The contextual standards covered the scope of ICT devices used in health, applications, networking and connectivity, security and privacy and the resources required for these components to function well-connected and secure. We argue that countries with health system challenges can adopt the same set of contextualised DHCI standards similar to that of Uganda. In Uganda’s case, to ensure mandatory standards are widely implemented, the MoH should engage implementation partners and all entities who wish to participate in the connected health system to collaborative plan cooperation on a phased implementation and subsequent monitoring of compliance with the standards. Although this study used potential DHCI users and DH stakeholders in Uganda to validate the contextualised standards, it is necessary to evaluate them after implementation. We, therefore, recommend that the standards be piloted to assess their potency to improve DHCI implementations that guarantee HIE.

## Supporting information

S1 Table Requirements for the Digital Health Communication Infrastructure to Support Health Information Exchange in Uganda. (DOCX)

S2 Table Statement and Rationale for Contextualised Digital Health Communication Infrastructure Standards for Uganda. (DOCX)

S3 Table Example of how to Apply Criteria for Selecting Candidate Standards to be Contextualised. (DOCX)

## References

[1] African Union. African Union Health Information Exchange Guidelines and Standards [Internet]. 2023 [cited 2023 Apr 2]. p. 108. Available from: https://africacdc.org/download/african-union-health-information-exchange-guidelines-and-standards/.

[2] Crichton R, Moodley D, Pillay A, Seebregts CJ. An interoperability architecture for the health information exchange in Rwanda. 2012;

[3] Ghana Ministry of Health. Ghana National eHealth Strategy. 2010.

[4] MoH-Uganda. Uganda National eHealth Policy and Strategy. 2016.

[5] Tanzania Ministry of Health. Tanzania National eHealth Strategy 2012–2018 [Internet]. 2013. Available from: https://www.who.int/goe/policies/countries/tza_ehealth.pdf.

[6] Union African. The digital transformation strategy for Africa (2020–2030) [Internet]. Addis Ababa: African Union; 2020. Available from: https://au.int/sites/default/files/documents/38507-doc-dts-english.pdf.

[7] MoH-Kenya. Standards and Guidelines for Electronic Medical Record System in Kenya. 2010;

[8] MoH-Sri Lanka. National eHealth Guidelines and Standards [Internet]. 2016. Available from: http://www.health.gov.lk/moh_final/english/public/elfinder/files/publications/list_publi/NeGS_v_1.pdf.

[9] Standards Australia. Publications: Standards Australia [Internet]. 2022 [cited 2022 Dec 22]. Available from: http://e-health.standards.org.au/Home/Publications.aspx.

[10] Mamuye A. L., Yilma T. M., Abdulwahab A., Broomhead S., Zondo P., Kyeng M. et al. Health information exchange policy and standards for digital health systems in africa: A systematic review. *PLOS Digital Health*. 2022;1:e0000118. doi: 10.1371/journal.pdig.0000118 36812615 PMC9931258

[11] ETSI. eHEALTH; Architecture; Analysis of user service models, technologies and applications supporting eHealth [Internet]. Sophia Antipolis Cedex—FRANCE; 2009 [cited 2021 Oct 26]. p. 48. Available from: http://www.etsi.org.

[12] ITU-T. Resolution 78 –Information and communication technology applications and standards for improved access to e-health services [Internet]. Hammamet; 2016. p. 5. Available from: https://www.itu.int/dms_pub/itu-t/opb/res/T-RES-T.78-2016-PDF-E.pdf.

[13] South African Department of Trade and Industry. National Norm for the development of South African National Standards [Internet]. SABS Standards Division; 2017. Available from: https://static.pmg.org.za/170421DraftNationalNormforthedevelopmentof_SANS.pdf.

[14] Liu H, Huffman MD, Trieu K. The role of contextualisation in enhancing non-communicable disease programmes and policy implementation to achieve health for all. *Health Research Policy and Systems*. 2020;18:38. doi: 10.1186/s12961-020-00553-5 32303249 PMC7164194

[15] Alunyu AE, Ssekibuule R, Nabukenya J. Towards Adoption of Standards for Communication Infrastructure/Technologies in Healthcare Systems in LMICs: Theories, Practice and Evaluation. *13th International Joint Conference on Biomedical Engineering Systems and Technologies (BIOSTEC 2020)*. *SCITEPRESS*; 2020. p. 735–744.

[16] Alunyu AE, Nabukenya J. A Conceptual Model for Adaptation of eHealth Standards by Low and Middle-Income Countries. *J Health Inform Afr*. 2018;5:10–16.

[17] Council of the European Union. List of standards and/or specifications for electronic communications networks, services and associated facilities and services [Internet]. 2004. p. 144. Available from: https://www.etsi.org/deliver/etsi_sr/002200_002299/002211/01.01.01_60/sr_002211v010101p.pdf.

[18] Choi DG, Kang BG, Kim T. Standardization: Fundamentals, Impact, and Business Strategy. Asia Pacific Economic Cooperation Sub-Committee on Standards and Conformance; 2010.

[19] ITU-T. ITU-T Recommendation I.130: Method for the characterization of telecommunication services supported by an ISDN and network capabilities of an ISDN [Internet]. 1988 [cited 2020 Jun 24]. Available from: https://www.itu.int/rec/T-REC-I.130-198811-I.

[20] MoH-Uganda. Strengthening Uganda’s Health System through Standardizing Digital Health: Requirements for Digital Health Standards and Enterprise Architecture Framework [Internet]. 1st ed. Kampala; 2021 [cited 2022 Nov 22]. Available from: http://library.health.go.ug/health-information-systems/digital-health/strengthening-ugandas-health-system-through-standardizing.

[21] Kiwanuka, A., Bagyendera, M., Wamema, J., Alunyu, A., Amiyo, M., Kambugu, A., et al. Establishing the State of Practice about Data Standards in Monitoring Healthcare Interventions for HIV in Uganda’s EMR-based Health Information Systems. *14th International Conference on Health Informatics*. *Vol 5*: *HEALTHINF*. *SCITEPRESS*; 2021. p. 200–211.

[22] McCann D. A Ugandan mHealth Moratorium Is a Good Thing [Internet]. 2012 [cited 2018 Aug 27]. Available from: https://www.ictworks.org/ugandan-mhealth-moratorium-good-thing/.

[23] Huang F, Blaschke S, Lucas H. Beyond pilotitis: taking digital health interventions to the national level in China and Uganda. *Globalization and Health*. 2017;13:49. doi: 10.1186/s12992-017-0275-z 28756767 PMC5535287

[24] Adebesin F, Kotze P, Foster R, Van Greunen D. A Review of Interoperability Standards in E-health and Imperatives for their Adoption in Africa. *South African Computer Journal*. 2013;50:55–72.

[25] Hammond WE. Standards for Global Health Information Systems. Academic Press. 2017;94–108.

[26] ITU-T. E-health Standards and Interoperability [Internet]. 2012. Available from: https://www.itu.int/dms_pub/itu-t/oth/23/01/T23010000170001PDFE.pdf.

[27] Serrano-Santoyo A, Rojas-Mendizabal V. E-Health standardization challenges in emerging economies: The case of Mexico. *ITU Kaleidoscope Academic Conference*: *Living in a converged world-Impossible without standards*?, *Proceedings of the 2014 [Internet]*. IEEE; 2014 [cited 2017 Oct 7]. p. 129–133. Available from: http://ieeexplore.ieee.org/abstract/document/6858489/.

[28] ESTI. A Guide to Writing World Class Standards [Internet]. 2013. Available from: https://portal.etsi.org/Portals/0/TBpages/edithelp/Docs/AGuideToWritingWorldClassStandards.pdf.

[29] HIPAA. HIPAA Security Rule Standards and Implementation Specifications [Internet]. HHS.gov. 2013 [cited 2021 Jul 12]. Available from: https://www.hhs.gov/hipaa/for-professionals/security/laws-regulations/index.html.

[30] ISO/IEC. ISO/IEC Directives, Part 2 Principles and rules for the structure and drafting of ISO and IEC documents [Internet]. 2021. Available from: www.iso.org.

[31] US Office of the National Coordinator for Health IT. Interoperability Standards Advisory: Best Available Standards and Implementation Specifications. 2015.

[32] Currie W. Contextualising the IT artefact: towards a wider research agenda for IS using institutional theory. Shoib G, editor. *Information Technology & People*. 2009;22:63–77.

[33] Ministry of ICT-Uganda. Data Protection and Privacy Act, 2019. 2019.

[34] Villalba-Mora E, Casas I, Lupiañez-Villanueva F, Maghiros I. Adoption of health information technologies by physicians for clinical practice: the Andalusian case. *International journal of medical informatics*. 2015;84:477–485. doi: 10.1016/j.ijmedinf.2015.03.002 25823578

[35] Delponte L, Grigolini M, Moroni A, Vignetti S, Claps M, Giguashvili N. ICT in the developing world [Internet]. 2015. Available from: http://publications.europa.eu/resource/cellar/527ad528-e51a-11e5-8a50-01aa75ed71a1.0001.02/DOC_1.

[36] Community Ciena. The Need for Speed in Healthcare Networks [Internet]. 2017 [cited 2019 Sep 2]. Available from: https://media.ciena.com/documents/The_Need_For_Speed_In_Healthcare_Networks_AN.pdf.

[37] Martinez I, Garcia J, Viruete E, Fernandez J. Performance Evaluation of Rural e-Health Scenarios: Users and QoS Management. *2006* *International Conference of the IEEE Engineering in Medicine and Biology Society*. 2006. p. 5234–5237. 10.1109/IEMBS.2006.259346 17946293

[38] Yang H, Qi L, Weirong C. Microgrid communication system and its application in hierarchical control. 2019. p. 179–204.

[39] Ensign B, Ortronics L, Oliver CE. TIA-1179 Healthcare Infrastructure Standard [Internet]. Cabling Installation & Maintenance. 2010 [cited 2019 Jun 19]. Available from: https://www.cablinginstall.com/standards/cabling-standards/article/16465083/tia1179-addresses-cabling-in-healthcare-facilities.

[40] McLaughlin P. Planning communications infrastructure for a healthcare facility [Internet]. 2018 [cited 2019 Feb 5]. Available from: https://www.cablinginstall.com/articles/print/volume-26/issue-4/features/design/planning-communications-infrastructure-for-a-healthcare-facility.html.

[41] South African National Department of Health. National Health Normative Standards Framework for Interoperability in eHealth in South Africa [Internet]. 2014 [cited 2019 Jun 11]. Available from: http://www.samed.org.za/Filemanager/userfiles/hnsf-complete-version.pdf.

[42] Kiberu VM, Mars M, Scott RE. Barriers and opportunities to implementation of sustainable e-Health programmes in Uganda: A literature review. *African Journal of Primary Health Care & Family Medicine*. 2017;9:1–10. doi: 10.4102/phcfm.v9i1.1277 28582996 PMC5458569

[43] Lima-Toivanen M, Pereira RM. The contribution of eHealth in closing gaps in primary health care in selected countries of Latin America and the Caribbean. *Revista Panamericana de Salud Pública*. 2019;42:e188. 10.26633/RPSP.2018.188 PMC6386043 31093215

[44] Quddusi MA. eHealth Technologies: The Benefits and Challenges of e-Health Applications [Internet]. The Scientific World—Let’s have a moment of science. 2019 [cited 2022 Sep 9]. Available from: http://www.scientificworldinfo.com/2019/09/the-benefits-and-challenges-of-e-health-technologies.html.

[45] Gavrilov G, Vlahu-Gjorgievska E, Trajkovik V. Healthcare data warehouse system supporting cross-border interoperability. *Health informatics journal*. 2020;26:1321–1332. doi: 10.1177/1460458219876793 31581924

[46] Altamimi AM. Security and privacy issues in eHealthcare systems: Towards trusted services. *International Journal of Advanced Computer Science and Applications*. 2016;7:229–236.

[47] Al-Issa Y, Ottom MA, Tamrawi A. eHealth cloud security challenges: a survey. *Journal of healthcare engineering*. 2019;2019. doi: 10.1155/2019/7516035 31565209 PMC6745146

[48] William C. and Security: Privacy of Personal eHealth Data in Low-and Middle-Income Countries. Global health informatics: principles of EHealth and MHealth to improve quality of care. 2017;269.

[49] Barakat A, Woolrych RD, Sixsmith A, Kearns WD, Kort HS. eHealth Technology Competencies for Health Professionals Working in Home Care to Support Older Adults to Age in Place: Outcomes of a Two-Day Collaborative Workshop. 2013 [cited 2018 Nov 4]; Available from: https://www.ncbi.nlm.nih.gov/pmc/articles/PMC4084768/. doi: 10.2196/med20.2711 25075233 PMC4084768

[50] Brice S, Almond H. Health professional digital capabilities frameworks: a scoping review. *Journal of multidisciplinary healthcare*. 2020;13:1375. doi: 10.2147/JMDH.S269412 33173300 PMC7646414

[51] Chan CV, Kaufman DR. A framework for characterizing eHealth literacy demands and barriers. *Journal of medical Internet research*. 2011;13:e1750. doi: 10.2196/jmir.1750 22094891 PMC3222196

[52] Nazeha N, Pavagadhi D, Kyaw BM, Car J, Jimenez G, Car LT. A digitally competent health workforce: scoping review of educational frameworks. *Journal of medical Internet research*. 2020;22:e22706. doi: 10.2196/22706 33151152 PMC7677019

[53] Cybernet. Medical Computers: Understanding What is Required in a Hospital Setting [Internet]. Requirements-medical-computers-hospital. 2020 [cited 2022 Sep 9]. Available from: https://www.cybernetman.com/blog/requirements-medical-computers-hospital/.

[54] Walugembe DR, Sibbald S, Le Ber MJ, Kothari A. Sustainability of public health interventions: where are the gaps? *Health Research Policy and Systems*. 2019;17:8. doi: 10.1186/s12961-018-0405-y 30646911 PMC6334403

[55] Kimaro H. Strategies for Developing Human Resource Capacity to Support Sustainability of ICT Based Health Information Systems: A Case Study from Tanzania. *EJISDC The Electronic Journal on Information Systems in Developing Countries*. 2006;26:1–23.

[56] Rowlands D. A Health Interoperability Standards Development, Maintenance and Management Model for Australia. 2020.

