# Supplementary material for: Contextualised digital health communication infrastructure standards for resource-constrained settings: Perception of digital health stakeholders regarding suitability for Uganda’s health system
Source: PLOS Digit Health. 2024 Sep 12;3(9):e0000603. doi: 10.1371/journal.pdig.0000603 (PMC11392385; doi:10.1371/journal.pdig.0000603)
Supplement: S1 Table — (DOCX) [file pdig.0000603.s001.docx]

# S1 Table

| Requirements for DHCI Standards to Support HIE in Uganda | Responses | | |
| --- | --- | --- | --- |
|  | **Agree** | **Disagree** | **Others** |
| Gap-01: MoH has not officially adopted any DH standards for the electronic sharing and exchange of patient data despite some health centres already implementing them |  |  |  |
| REQ_01: The MoH should officially adopt DHCI standards to support HIE | | | |
| R1_RS1: A readiness assessment of healthcare points/sites should be done to ascertain DH preparedness and stipulate critical areas of infrastructure to be funded and the responsible authorities | 99% | 1% | 0% |
| R1_RS2: The standards should be explicit about the nature of data /health information exchange across the applications and health facilities, including the role of human resources in protecting the data while using DHCI | 99% | 0% | 1% |
| R1_RS3: The standards should be implementable using the available minimum resources (technical expertise, technology, infrastructure, finance etc.) | 99% | 1% | 0% |
| R1_RS4: The standards should guide on how critical areas and/or changes in the DHCI can be identified and funded | 99% | 0% | 1% |
| R1_RS4: The standards should document what MoH and related MDAs have specified as minimum devices requirements to guide HIV/TB healthcare sites, organisations and agencies in acquiring ICT devices for digital health; and these should be contextual, ubiquitous and secure. | 100% | 0% | 0% |
| R1_RS5: The standard should specify potential methods for funding to ensure the sustainability of the DHCI | 98% | 2% | 0% |
| REQ_02: The MoH should develop minimum requirements/guideline for health facilities and agencies for acquiring and establishing communication infrastructure for DH that is contextual, ubiquitous and secure to enable the protection, privacy and confidentiality of shared data | | | |
| R2_RS1: The minimum requirements should specify topologies, protocols, middleware, security and privacy mechanisms | 97% | % | 3% |
| R2_RS2: The guideline for HIV/TB DHCI's communication networks for Uganda should specify the type of networks, cabling, and data centre/backup storage that ensure support for reliable delivery of patient centred-care across the different points of care. | 98% | 2% | 0% |
| R2_RS3: The minimum requirements should specify a set of administrative, technical and managerial actions that should be applied during the lifecycle of the eHealth infrastructure | 97% | 2% | 1% |
| R2_RS4: Any MoUs with telecommunication service providers in Uganda should specify terms for zero-rating for services that support HIV/TB patient care and data communications. | 100% | 0% | 0% |
| R2_RS5: Any MoUs should specify the expected quality of service to support HIV/TB programs and healthcare communication services in Uganda's healthcare system | 100% | 0% | 0% |
| REQ_03: The MoH and related MDAs should develop a standard on HIV/TB DH applications designing and development to aid integration with different electronic records and information systems to ease seamless sharing of patient health data | | | |
| R3_RS1: The standard should stipulate mechanisms for regulating DH applications for HIV/TB services through testing and evaluating them before adopting them for use in Uganda. | 100% | 0% | 0% |
| R3_RS2: The guidelines should specify a set of administrative, technical and managerial actions that should be applied during the lifecycle of the DH application for HIV/TB and the data that they hold | 98% | 2% | 0% |
| R3_RS3: MoH should stipulate minimal system and runtime software requirements to run on DH hardware and devices | 97% | 1% | 2% |
| R4_RS4: MoH Should enumerate ICT technologies to be used in healthcare |  |  |  |
| REQ-04: MoH should officially adopt DH security policies, standards and guidelines | | | |
| R4_RS1: The security and privacy standard should be tailored towards the eHealth policy and /strategy for Uganda as well as the Data Protection and Privacy Act 2019 and international information security standards such as ISO/IEC 2700 series, among others; outlining how the cardinal principles of confidentiality, integrity, and availability (CIA) should guide policies/controls designed to protect health data | 100% | 0% | 0% |
| R4_RS2: The standard should define the full scope of eHealth security and data privacy measures that go beyond physical security to identifying health information and related assets, plus potential threats, vulnerabilities and impacts | 100% | 0% | 0% |
| R4_RS3: The security and privacy standard should have clear indicators for compliance with the Data Protection and Privacy Act and specify a mechanism for monitoring implementation and enforcing compliance with DHCI and HIV/TB data security controls | 98% | 2% | 0% |
| R4_RS4: The MoH and healthcare organisations should sensitise and or train stakeholders on existing standards for security and privacy so that users can comply with existing security laws and procedures aimed at protecting HIV/TB Applications, technologies, and data | 99% | 0% | 1% |
| R4_RS5: The security and privacy standard should have a plan specifying how risks faced by DHCI and data residing on them can be mitigated. In addition, the plan should specify a mechanism for monitoring implementation and enforcing compliance of DHCI with health data security controls | 99% | 0% | 1% |
| R4_RS6: The standard should stipulate human resources skills required to securely use the DH systems (applications, software, technologies) to protect health data | 98% | 2% | 0% |
| Gap-02: Inadequacy of the reviewed/ updated/ contextualised standards |  |  |  |
| RS1: The MoH should periodically review/ update DHCI standards to suit Uganda's context | 97% | 1% | 2% |
| RS2: The contextual DH standards should provide for technical and implementation specifications for all domains of the DHCI, i.e., hardware, software, applications, communication network, security and privacy, and other facilitating resources of the DHCI environment | 99% | 0% | 1% |
| Gap-03: Poor documentation/ expression standards make them complex and difficult to understand, which leads to inadequacy of the reviewed/ updated standards to be implemented in a way that meets its desired requirements | 99% | 1% | 0% |
| RS1: The MoH should develop DHCI standards in simple language, structure, and format with clear guidelines. | 99% | 0% | 1% |
| RS2: Expression of the standards should be clear with simple language, structure, and format that state the characteristics/ benefits/ rationale of implementing it |  |  |  |
| RS3: The standards should be well documented | 100% | 0% | 0% |
| Gap-04: Inconsistent presentation of the standards |  |  |  |
| RS1: MoH should adopt a structure and format of expressing standards to improve implementer understanding --- Same as in Gap-03 | 99% | 0% | 1% |
| Gap-05: Inadequate resources to support standardisation |  |  |  |
| RS1: The standards should specify the nature of formal pre-service training curricula /training programmes that students should undertake to qualify with min. required to implement DHCI standards and use DH applications/technologies | 98% | 2% | 0% |
| RS2: The standards should stipulate what in-service training programmes for DH workers should be addressed | 99% | 0% | 1% |
| RS3: The standards should specify critical areas of infrastructure that support HIV/TB to be funded, possible sources of funding and the responsible authorities | 99% | 1% | 0% |
| RS4: The standard should emphasise the use of diverse sources of energy, focusing on clean energy (renewable energy) such as solar, wind, water (hydro), biomass, and geothermal. | 98% | 1% | 1% |
