# Supplementary material for: Contextualised digital health communication infrastructure standards for resource-constrained settings: Perception of digital health stakeholders regarding suitability for Uganda’s health system
Source: PLOS Digit Health. 2024 Sep 12;3(9):e0000603. doi: 10.1371/journal.pdig.0000603 (PMC11392385; doi:10.1371/journal.pdig.0000603)
Supplement: S2 Table — (DOCX) [file pdig.0000603.s002.docx]

# S2 Table

| **Contextualised standard for Uganda's DHCI to support HIV/TB Health Information Exchange for Patient-centred care** |
| --- |
| 1. **DH Hardware Devices (**ANSI/BICSI & Institute, 2018; ISO/IEEE, n.d.; ISO/TR, 2009; TR-42.1, 2017; MoH’s Device Specification, work in progress; NITA-U, 2013; Singapore Ministry of Health, 2015; The Open Group, 2018) |
| **1.1 Align DHCI Standard Implementations**  Critical areas and or changes in the DHCI are appropriately aligned to the country's digital health needs, eHealth strategy, and related policies.  ***Rationale***  **1.1.1** Changes in the DHCI are continuously researched, guided and well documented based on current DH user needs. |
| **1.2 DHCI lie within the country’s DH technology blueprint**  Minimum specifications of HW devices restrict implementations of the DHCI within the country's DH technology blueprint that assures healthcare facilities/ organisations participating in the connected health system establish a DH communication network with quality devices, data rate, and network services.  ***Rationale***  **1.2.1** Hardware devices should be able to support all forms of approved DH applications, health data and information being captured, processed, presented and or shared.  **1.2.2** Software running on the devices should be licensed and support installation (be compatible) of other DH applications |
| **1.3 Minimise diverse implementation of the DHCI**  Reduce diversity of DHCI implementations that support healthcare programmes and interventions through procurement and implementation based on contextualised global standards and min. specifications adopted/laid down by MoH and related MDAs.  ***Rationale***  1.3.1 The MoH enforces implementation and use of interoperable DHCI and applications capable of facilitating exchange of health data/information across all health system levels. |
| 1. **Communication Networks & Connectivity** (Australian Commission on Safety, 2011; European Parliament, n.d.; European Union, 2015; HIPAA, 2013; ITU-T, 2015; MoH’s Device Specification, work in progress; The Open Group, 2018; TR-42.1, 2017, p.) |
| **2.1 Standardised Connected Health Systems**  Healthcare facilities/ organisations participating in the connected health system should establish DH communication network(s) with quality devices, data rate, & suitable network services to support secure HIE.  **Rationale**  **2.1.1** All participating health organisation/ facilities implement DHCI that meet the required hardware, software, network and security specifications for quality health data. |
| **2.2 MoU Specifying Telecom and Internet service Provision**  Any form of MoU and SLA (as part of the social corporate responsibility) between with telecom and Internet service providers should specify both quality and terms of service provision to Uganda's health sector.  **Rationale**  **2.2.1** There is reliable and affordable (subsidised) connectivity to support all forms of HIE in healthcare. |
| **2. 3 Last Mile Connectivity**  NITA-U should connect all health facilities to the National Backbone Infrastructure.  ***Rationale***  **2.3.1** Government Health facilities and Service delivery points are connected to the National Data Transmission Backbone Infrastructure. |
| 1. **DH Applications, Software and Technologies–** (European Union, 2015; Singapore Ministry of Health, 2015; The Open Group, 2018) |
| **3.1** **Application Design & Development**  DH application designing and development is guided by agreed standards and guidelines as contextualised by MoH and related MDAs, which specify modes for linking (sharing) health data between applications in a secure manner.  ***Rationale***  **3.1.1** Design, development, and deployment of DH applications are guided by standards that specify modes for linking (sharing) health data from across DH applications in a secure manner. |
| **3.2 Mandate to Oversee all DHCI Implementation**  MoH superintends the implementation and use of DH applications in public and private healthcare sites.  ***Rationale***  **3.2.1** The MoH has mechanisms for regulating development and deployment of DH applications for healthcare services in Uganda.  **3.2.2** MoH superintends DH infrastructure/ Applications implementation and use at both public and private health facilities. |
| **3.3 Up-to-date and Interoperable Software**  Both system and general application softwares that are run on DH devices have update security measures.  ***Rationale***  **3.3.1** Software running on the devices should be licenced and support installation (be compatible) of DH applications.  **3.3.2** MoH ensures that implementing entities run software that often provide upgrades including security updates. |
| **3.4 Acceptable DH Technologies**  Control choice of DH technologies to be adopted and used by healthcare organisations, facilities and researchers to ensure interoperable and secure collection, sharing and use of health data/information.  ***Rationale***  **3.4.1** MoH should determine and enlist acceptable (including emerging) ICT technologies that healthcare participating entities can adopt to support healthcare processes in Uganda. |
| 1. **Security, Privacy and Secure Use of the DHCI** (HHS Office of Civil Rights, 2013; Health IT, 2015; HIPAA, 2013; European Union, 2019; ITI Planning Committee, 2015; EHRS FM R2; ISO/HL710781; ISO IS 17090-1 2013; ISO IS 17090-2 2008; MoICT-Uganda, 2019; The Open Group, 2018) |
| **4.1** ***DH Information Security Access Rights***  DH information security access rights are properly assigned and managed.  ***Rationale***  **4.1.1** Appropriate information access and security clearance levels are assigned (based on the relevancy of their roles or duties) and managed for all members of the workforce and other authorised parties.  **4.2** ***DH Data/Information Transmission Security***  Develop policy-based guidelines for securing the DH data/information transmission.  ***Rationale***  **4.2.1** The MoH and related MDAs responsible for the DH standardisation have developed guidelines for data/information transmission within and between networks and devices.  **4.3 *Data Protection – Confidentiality and Integrity***  Enforce measures that provide for data/information confidentiality and protection are implemented and enforced across the entire health sector.  ***Rationale***  **4.3.1** The MoH and participating entities in the health sector ensure protection and privacy of personally identifiable data that the DHCI contains. |
| **4.4 Improve user observance of security and privacy guidelines**  Users are conversant and comply with DHCI relevant security and privacy laws, and guidelines including Uganda's Data Protection and Privacy Act regarding electronic data collection, processing, retention, and exchange.  ***Rationale***  **4.4.1** There is widespread sensitisation and monitoring to improve user compliance with DHCI relevant security and privacy laws, standards/guidelines. |
| **4.5 *Mitigation of security risks***  DH systems have mitigation plans addressing security risks faced by DHCI and the health data they contain  **Rationale**  **4.5.1** All entities in the health sector must mitigate, to the extent practicable, any harmful effect that may result from the violation of security policies and guidelines or the requirements. |
| 1. **Facilitating Resources (Technical & Skilled Human Resources, Financial Resources, and Electrical Energy to power the DH)** |
| **5.1 *Training of DH Users***  The MoH, collaborating medical training institutions and the DH implementing partners should aim at producing an all-rounded, knowledgeable and skilled digital health workforce capable of observing stipulated guidelines.  ***Rationale***  **5.1.1** Ensure all rounded, knowledgeable and skilled DH health workforce for improved service delivery. |
| **5.2 Training of Managers responsible for DHCI**  Leaders/managers responsible for DHCI are informed and trained on the use of DH technologies and trends.  ***Rationale***  **5.2.1** Leaders/managers responsible for DHCI are made aware of emerging technologies and trends to support their decision-making process. |
| **5.3 *DHCI Financing***  Adequate funds should be allocated to finance the DHCI that supports DH data collection, processing and information sharing in Uganda.  ***Rationale***  **5.3.1** Resources are identified and equitably allocated to the critical areas of the DHCI for efficient deployment of DH in a manner that enables the health sector levels (National & Subnational) acquire and implement the DHCI defined for the different levels by MoH. |
| **5.4 *Reliable Electric Power Supply***  Reliable and alternative sources of electric energy are available at all healthcare service delivery points.  ***Rationale***  **5.4.1** Whenever possible, DH systems are powered by electric connections to the national grid and or alternative smart sources of electric energy are made available at all health service delivery points. |
