# Supplementary material for: Contextualised digital health communication infrastructure standards for resource-constrained settings: Perception of digital health stakeholders regarding suitability for Uganda’s health system
Source: PLOS Digit Health. 2024 Sep 12;3(9):e0000603. doi: 10.1371/journal.pdig.0000603 (PMC11392385; doi:10.1371/journal.pdig.0000603)
Supplement: S3 Table — (DOCX) [file pdig.0000603.s003.docx]

# S3 Table

|  | **Domains of DHCI** | **Design & implementation pf DHCI Communication Network** | | | | **Security & Privacy standards** | | | | |
| --- | --- | --- | --- | --- | --- | --- | --- | --- | --- | --- |
| **Standards Selection Criteria** | **Relevant Standards for DHCI** | **ANSI/BICSI 004-2018** | **ANSI/AAMI SW68** | **ANSI/TIA-942** | **ANSI/TIA-1179-A** | **Uganda Inf Security** | **ISO/IEC 27033-4:2014** | **ISO 27799:2016** | **ISO/IEC 29100** | **HIPAA** |
| **General Criteria** | |  |  |  |  |  |  |  |  |  |
| Constrained/extended from existing IT standards | | 2 | 0 | 0 | 3 | 3 | 0 | 0 | 0 | 3 |
| Supports healthcare business requirements | | 0 | 0 | 0 | 1 | 1 | 0 | 0 | 0 | 1 |
| Supports technical requirements | | 3 | 3 | 3 | 3 | 3 | 3 | 3 | 3 | 3 |
| Adoption likelihood | | 2 | 3 | 3 | 3 | 3 | 3 | 3 | 3 | 3 |
| Supports coded data vs. free text | | 1 | 1 | 1 | 1 | 3 | 1 | 2 | 2 | 2 |
| Has governance and or administrative component | | 1 | 1 | 2 | 2 | 3 | 2 | 2 | 2 | 3 |
| Shared i0ntellectual property and licensing costs | | 0 | 0 | 0 | 0 | 0 | 0 | 0 | 0 | 0 |
| Defined maintenance process | | 2 | 2 | 2 | 2 | 3 | 2 | 2 | 2 | 2 |
| Cost of possible implementation are known | | 0 | 0 | 0 | 0 | 0 | 0 | 0 | 0 | 0 |
| Provides implementation support & education | | 1 | 1 | 1 | 1 | 2 | 1 | 1 | 1 | 1 |
| Enables/supports interoperability of DHCI systems | | 1 | 1 | 1 | 3 | 3 | 1 | 1 | 1 | 2 |
| Has implementation and maintenance tools | | 1 | 1 | 1 | 1 | 2 | 1 | 1 | 1 | 1 |
| Conformance testing methodologies and tools | | 0 | 0 | 0 | 1 | 2 | 1 | 1 | 1 | 2 |
| Proven stability | | 2 | 2 | 2 | 2 | 1 | 2 | 2 | 2 | 3 |
| Adaptable and customizable | | 3 | 3 | 3 | 3 | 3 | 3 | 3 | 3 | 3 |
| **DHCI Specific Criteria** | |  |  |  |  |  |  |  |  |  |
| It’s in sync with market trend & responsive to user needs | | 1 | 1 | 1 | 3 | 3 | 1 | 1 | 2 | 2 |
| Adaptable to DH technology blueprint of the adopter environment to control technology diversity | | 0 | 0 | 0 | 1 | 3 | 1 | 1 | 1 | 2 |
| Compatibility with other existing standards for DHCI e.g., cabling, work spaces, etc | | 3 | 3 | 3 | 3 | 2 | 3 | 3 | 3 | 3 |
| Specifies QoS/reliability parameters for healthcare | | 0 | 0 | 0 | 1 | 0 | 0 | 0 | 0 | 0 |
| Generic standards for DH applications independent of the technology | | 3 | 3 | 3 | 3 | 3 | 3 | 3 | 3 | 3 |
| It has a flexible implementation approach | | 1 | 1 | 1 | 1 | 1 | 2 | 2 | 2 | 2 |
| Implementation completeness - adequate to guide complete implementation of DHCI | | 2 | 2 | 2 | 3 | 2 | 2 | 2 | 2 | 3 |
| **Resource-Related Criteria** | |  |  |  |  |  |  |  |  |  |
| Adaptable to available Bandwidth in RC settings | | 0 | 0 | 1 | 1 | 1 | 2 | 2 | 2 | 2 |
| Adaptable to available Hardware | | 2 | 2 | 3 | 3 | 1 | 2 | 2 | 2 | 3 |
| Supports security and privacy needs of healthcare | | 3 | 3 | 3 | 3 | 3 | 3 | 3 | 3 | 3 |
| Supports availability & reliability of available mobile (ubiquitous) network connectivity | | 3 | 3 | 3 | 3 | 2 | 2 | 2 | 2 | 2 |
| Specifies needed ICT literacy or training needs | | 1 | 1 | 1 | 2 | 1 | 1 | 1 | 1 | 3 |
| Support use of alternative sources of electric energy/power | | 3 | 3 | 3 | 3 | 1 | 3 | 3 | 3 | 3 |
| **Total score per standards out of the expected140** | | **41** | **40** | **43** | **56** | **55** | **45** | **46** | **47** | **60** |
| **Key:** How to apply the selection criterion: To determine a suitable global standard for adoption/adaption, we score the performance of each standards against each criterion. The workgroup recommended a Linkert scale scoring system of 1-5 (1 = Poor, 2 = Fair, 3 = Good, 4 = Very Good, and 5 = Excellent). This means the highest total score that any standards could score is 140 (maximum score of 5 for each of the 28 selection metrices). Furthermore, they proposed that the grading of total score (used to determine the most suitable standard in each case) should be organised in the ranges for Poor/Bad to Excellent. However, standards can only be considered as either suitable or unsuitable for a particular application. Therefore, a standard with the highest score should be considers suitable for contextualisation but a standard with a total score of 140 are potential candidates for adoption ‘as is’. | | | | | | | | | | |
